# Clinical and Genetic Screening for Hypertrophic Cardiomyopathy in Paediatric Relatives: Changing Paradigms in Clinical Practice

**DOI:** 10.3390/jcm12082788

**Published:** 2023-04-09

**Authors:** Claire M. Lawley, Juan Pablo Kaski

**Affiliations:** 1 Centre for Inherited Cardiovascular Diseases, Great Ormond Street Hospital, London WC1N 3JH, UK; 2 The University of Sydney Children’s Hospital Westmead Clinical School, Faculty of Medicine and Health, The University of Sydney, Camperdown, NSW 2006, Australia; 3 Centre for Paediatric Inherited and Rare Cardiovascular Disease, University College London Institute of Cardiovascular Science, London WC1E 6DD, UK

**Keywords:** hypertrophic cardiomyopathy, paediatrics, genetics, precision medicine, screening, sudden death

## Abstract

Hypertrophic cardiomyopathy (HCM) is an important cause of morbidity and mortality in children. While the aetiology is heterogeneous, most cases are caused by variants in the genes encoding components of the cardiac sarcomere, which are inherited as an autosomal dominant trait. In recent years, there has been a paradigm shift in the role of clinical screening and predictive genetic testing in children with a first-degree relative with HCM, with the recognition that phenotypic expression can, and often does, manifest in young children and that familial disease in the paediatric age group may not be benign. The care of the child and family affected by HCM relies on a multidisciplinary team, with a key role for genomics. This review article summarises current evidence in clinical and genetic screening for hypertrophic cardiomyopathy in paediatric relatives and highlights aspects that remain to be resolved.

## 1. Introduction

Hypertrophic cardiomyopathy (HCM) is a clinically and genetically heterogeneous disease characterised by unexplained left ventricular hypertrophy (LVH). While the estimated population prevalence in adults is 1 in 500, it is substantially rarer in the paediatric population, with a reported prevalence of 2.9/100,000 [1] and an estimated annual incidence of 0.3–0.5 per 100.000 [2,3]. A childhood HCM diagnosis may follow a review for cardiac symptoms or the incidental finding of a murmur or an electrocardiogram (ECG) abnormality, or, increasingly, as a result of family screening.

The outcomes of childhood-onset HCM are influenced by aetiology and age at diagnosis. Children diagnosed during infancy or with an inborn error of metabolism (IEM) have a worse prognosis, with a recent study reporting a 5 year survival rate of 80.5% in those diagnosed aged less than one year and 66.4% in those with an IEM at a median of just over 5 years follow-up [4]. In two cohorts with infant diagnoses of HCM, survival beyond the first year was associated with a more favourable natural history [5,6]. In a consortium study including 639 children diagnosed younger than 12 years with non-sarcomeric HCM, almost 10% experienced death or cardiac transplantation, and almost 11% experienced a life-threatening arrhythmic event in a median follow-up of 5.6 years [7]. Importantly, over 50% had a family history of HCM and only 20% were symptomatic, suggesting that adverse outcomes in childhood-onset HCM are not limited to symptomatic probands. Furthermore, children with HCM diagnosed in childhood have been shown to carry a higher risk of life-threatening ventricular arrhythmias and have a greater need for advanced heart failure therapies when compared to those diagnosed in adulthood [8]. Data from the Sarcomeric Human Cardiomyopathy Registry (SHaRe) identified that childhood-onset HCM patients had roughly a 2% per year event rate of serious adverse events, including ventricular arrhythmia, heart failure, and atrial fibrillation, as well as stroke and death, with ventricular arrhythmia being the most common of these adverse events in the first decade following baseline review. From the same registry, work including 4591 individuals with HCM showed that those with pathogenic/likely pathogenic variants in sarcomeric genes had a 2-fold greater risk of adverse outcomes, and those diagnosed before the age of <40 (including 422 paediatric patients) had a higher incidence of the overall composite outcome than those diagnosed at a later age [9].

**Figure 1:**
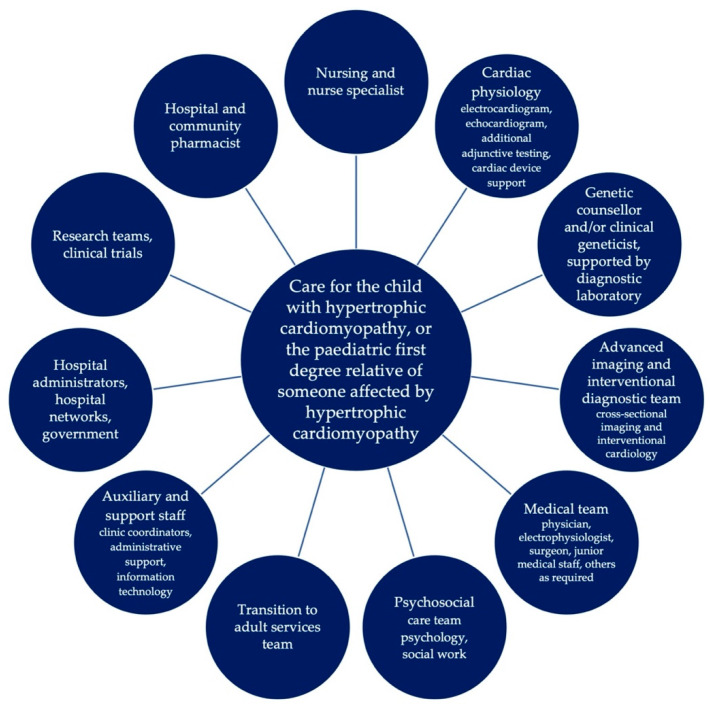
A proposed multidisciplinary approach to caring for the child or adolescent with HCM or a paediatric first-degree relative of someone affected by HCM.

As in adults, the management of children with HCM and paediatric first-degree relatives of individuals with HCM requires a multidisciplinary team approach. Figure 1 identifies some of the relevant medical teams involved in the care of children and families affected by HCM. Holistic care includes not only symptom management and prevention of disease-related complications (particularly sudden cardiac death) in affected individuals but also well-supported family screening.

In recent years, there have been important advances in the aetiology-specific management of HCM, particularly in childhood. In infants with HCM in the setting of Pompe disease, a glycogen storage disorder, enzyme replacement therapy has been shown to reduce the degree of LVH and may have a major impact on the natural history of this condition [10]. Similarly, MEK inhibitors are showing some promise in modifying ventricular hypertrophy in infants and children with HCM and a genetic variant in the Ras/MAPK cell signalling pathway (e.g., Noonan syndrome and related disorders) [11,12]. Cardiac myosin inhibitors (mavacamten and the next drug in the class, aficamten) have been shown to improve symptoms and reduce left ventricular outflow tract obstruction in adults with symptomatic obstructive HCM [13,14,15]. Preclinical studies suggest that their use in mice with sarcomeric gene variants without phenotypic evidence of LVH may prevent the development of the disease [16]. Finally, genetic modification in monogenic HCM is being explored; gene repair in human embryos with a pathogenic *MYBPC3* variant has been shown to be feasible using a variant-specific CRISPR-Cas9 system [17], and ‘gene silencing’ techniques, initially focusing on *MYH7* variant HCM and using viral vectors, are also being investigated [18,19].

## 2. Aetiology of Childhood HCM

The aetiology of childhood-onset HCM is heterogeneous and includes cases with genetic variants affecting the Ras/MAPK cell signalling pathway, inborn errors of metabolism (IEM) (including storage disorders), and neuromuscular diseases such as Friedreich’s ataxia [6,20]. However, recent data have shown that, in the majority of children with HCM, as in adults, the disease is caused by variants in the sarcomere protein genes, inherited as an autosomal dominant trait [9,21,22,23]. The list of implicated genetic variants in childhood-onset HCM continues to expand, and there are data to suggest that rates of ‘actionable’ variants may vary by age of onset of disease and ancestry [21]. Broad categories of paediatric HCM are outlined in Table 1.

## 3. Screening during Childhood and Adolescence for Those with an Affected First Degree Relative

Traditionally, HCM has been considered a disease of late adolescence and young adulthood. Until very recently, clinical practise guidelines suggested that, in the absence of symptoms, family history of premature death from HCM, competitive athletics, or other clinical suspicion of early LV hypertrophy, children of first-degree relatives with HCM need not be under regular screening until after 10 to 12 years of age [30,31,32]. Familial HCM was rarely reported in the paediatric literature, with data limited to small series, and there was an assertion that LVH did not develop until adolescence in patients with familial disease [33,34]. It was also felt that diagnosis earlier in childhood would not change outcome [32,35] and that earlier screening would have the potential to increase familial anxiety without any decrease in morbidity or mortality associated with an earlier diagnosis.

More recent data, however, have challenged the paradigm of delayed screening. A large single-centre study of 1198 consecutive children aged 18 years or less with a first-degree relative with HCM referred for clinical screening showed that in almost 10% of families, diagnostic criteria for HCM were met at the first or subsequent evaluation. The majority of these children presented as preadolescents, with a median age at diagnosis of 10 years, including a substantial proportion in infancy [36]. Importantly, the diagnosis of HCM changed the clinical course in nearly one-third of these children, including starting medication for symptoms that previously may have been attributed to non-cardiac disease and proceeding to surgery or an implantable cardiac device. Although patients with a diagnosis made through screening in childhood were more likely to have a family history of childhood disease, this only accounted for half of patients with early-onset disease, and neither the genetic nor the clinical features of those in whom an early diagnosis was made were different from those without childhood-onset disease. These findings were validated in an independent North American cohort, showing that 50% of individuals under the age of 18 diagnosed with HCM during screening were aged less than ten years and that 41% of the major cardiac events occurred in children aged less than ten years. Approximately one-third of the children diagnosed as a result of family screening would not have been screened under the historic guidelines [37]. In addition, patients with disease-causing variants in *MYH7* and *MYBC3* were at the highest risk for developing HCM in childhood and experiencing an adverse event or requiring intervention. Indeed, while previous data had suggested that *MYBPC3* variants in particular were associated with late-onset disease [38], more recent studies have shown that *MYBPC3* variants in heterozygosis can cause severe phenotypic expression of disease in childhood, with a high prevalence of malignant ventricular arrhythmia [39]. Furthermore, there are data to show that, in the context of a multidisciplinary expert setting and with adequate psychological support, clinical screening of paediatric first-degree relatives does not result in psychological harm [40].

The most recent American Heart Association/American College of Cardiology Guidelines for the Diagnosis and Treatment of Patients With Hypertrophic Cardiomyopathy now suggest that clinical screening for HCM should be offered to all first-degree relatives at the time at which the diagnosis is made in the family, regardless of age [41]. In support of this approach, data are also emerging for other cardiomyopathy subtypes, including arrhythmogenic right ventricular cardiomyopathy (ARVC). A recent single-centre cohort study of consecutive ARVC probands and genotype-positive relatives aged ≤18 years found that 40% met diagnostic criteria by age <12 years and that half of the cardiac adverse events occurred in these children [42]. Together, these data herald a need for a screening paradigm shift with the recognition that significant disease exists in pre-adolescents with genetic cardiomyopathies [43].

### 3.1. The Role of Predictive Genetic Testing in Children in Familial Screening for HCM

In families in which a ‘causative’ genetic variant (i.e., ‘pathogenic’ or ‘likely pathogenic’ variant) has been identified, predictive genetic testing of pre-phenotypic first-degree relatives should be considered, including in children [41]. When considering embarking on genetic testing for HCM in eligible families, pre- and post-test genetic counselling is essential [41]. In the clinically unaffected child, a ‘negative’ genetic test (whereby the child is found not to carry the familial causative gene variant) offers the opportunity to discharge the child from regular clinical follow-up, placing them at most at the general population risk of developing HCM. A ‘positive’ genetic test (whereby the child is found to carry the familial causative gene variant) confers a greater lifetime risk of developing HCM. Providing a personal estimate of the lifetime risk of HCM in these individuals remains fraught, but it is estimated that up to 50% of phenotype-negative individuals with disease-causing variants in the sarcomere protein will develop clinical disease within 15 years [44]. Crucially, while some clinical features, such as ECG abnormalities, are predictive of subsequent phenotype development, age at screening is not, and the penetrance of sarcomeric disease is the same regardless of the age at which an individual is assessed.

There are multiple factors that may influence a family’s or child’s decision to undergo predictive genetic testing. Families may wish to be equipped with the knowledge of who is at a higher risk of HCM or who can be discharged. While not quantified, the family’s experience with HCM to date may be a critical factor; severe disease in younger family members may serve as both a reason to test and a psychological deterrent to testing, for example, if there is a preference ‘not to know’. Genetic testing may allow for timelier clinical cascade testing, identifying other at-risk individuals who would not previously have been screened. Family preference and societal and cultural norms around consent and parental responsibility may also play a part in determining at what age genetic testing is undertaken. Some families may feel a duty to allow a child to be old enough to ‘choose’ to have genetic testing, versus the desire or perceived benefit of having a genetic result prior to this age in the best interests of the child [45]. Some health systems only offer pre-implantation genetic diagnosis for HCM to couples where one member has HCM and they do not already have a previously ‘unaffected’ child [46]. In such instances, while ethically uncertain, this may also serve as a motivator to undertake genetic testing in childhood. Genetic testing in children has been shown, at least in the short term, not to be deleterious to a child’s mental health [47,48,49].

### 3.2. Challenges and ‘the Unknowns’ in Familial Screening in Childhood for HCM

While there is now good evidence to support offering clinical and genetic screening to paediatric first-degree relatives of individuals with HCM, uncertainties still remain. Recent data in adult cohorts suggest that in patients with non-genetic HCM, the disease is less likely to be familial [50,51], but whether the same applies in paediatric HCM is unknown. Factors influencing the penetrance of any monogenic variant identified in childhood remain enigmatic. Although there are established monogenic contributions to disease, with mendelian inheritance, penetrance is variable; there are individuals who are identified to carry the familial causative (‘pathogenic’ or ‘likely pathogenic’) genetic variant who may never develop a clinical phenotype or only a mild clinical phenotype at a later age [52]. With associated genome-wide studies, the development of polygenic predictors continues to evolve [53]. Increasingly, the role of non-genetic factors as possible contributors to phenotypic expression is being better understood [54]. In adults, well-established risk factors associated with incident HCM include hypertension and obesity. Whether or not these play a role in childhood expression remains to be seen.

Further, in a study by Norrish et al., in those children in whom a childhood diagnosis of HCM was made as a result of family screening, the diagnosis was made during the first clinical visit in 56% of cases, suggesting that ongoing screening, in the context of age-related penetrance, is warranted. The frequency and nature of repeat clinical screenings remain to be determined. It has been shown that penetrance of sarcomeric variants is higher in the presence of an abnormal ECG at the initial screening visit, including in children [44], and more studies are needed to determine if there are additional predictors of subsequent phenotypic expression. Finally, while it is clear that childhood-onset familial HCM exists and that it is not necessarily a benign disease, warranting screening in childhood [36], as in other genetic cardiomyopathies, the survival benefit conferred by an earlier diagnosis needs to be studied systematically [43].

## 4. Conclusions and Future Directions

In the last decade, data from population-based studies and large international consortia of paediatric HCM have led to an improved understanding of its clinical presentation and natural history. With this has come a recognition that phenotypic expression can, and often does, occur in young children and that early diagnosis is possible with systematic clinical screening strategies. The age-old medical edict of ‘first do no harm’ must of course underpin the rationale for screening paediatric first-degree relatives of individuals with HCM, with the ultimate aim of identifying children with a potentially life-altering or threatening diagnosis earlier to allow intervention both for the individual and others who would then be identified as at risk through cascade testing. The delicate balance of this goal against the potential harms to individuals, families, and health systems needs to be considered. Current evidence suggests that, in the context of expert multidisciplinary services, clinical and genetic cascade screening should be offered to all children with a first-degree relative with HCM, regardless of their age. Ensuring equitable access to this multidisciplinary care, including genetic and psychological support for children and families affected by HCM, is paramount.

## Figures and Tables

**Table 1:** An approach to classifying paediatric hypertrophic cardiomyopathy.

Category	Example Conditions	Cardiac Features	The Most Common Mode of Inheritance	Example Candidate Genes
“Sarcomeric” HCM [9,22,24,25]	“Thick filament” disease “Thin filament” disease Other	Variable hypertrophy, arrhythmic risk, and patterns of hypertrophy with some genotype-phenotype correlation	Autosomal dominant *	*MYH7*, *MYBPC3* *TNNI3*, *TNNT2*, *TPM1*, *MYL2*, *MYL3*, *ACTC1*, *TNNC1* CSPR3, FLNC ** Additional noncoding variants influencing phenotype
Non-sarcomeric HCM				
Ras/MAPK disease [26]	Noonan syndrome, Costello syndrome, and cardiofaciocutaneous syndrome	Polyvalvulopathy, biventricular hypertrophy, infancy/early childhood presentation	Autosomal dominant	*RAF1, RIT1, PTPN1, HRAS, MEK1, MEK2*
Inborn errors of metabolism [27,28]	Glycogen storage disorders (Pompe disease, Danon disease, Cori-Forbes disease, and PRKAG2 syndrome) Lysosomal storage disorders (mucopolysaccharidoses)	Disease specific; specific ECG changes (such as short PR, ventricular pre-excitation), variable hypertrophy, polyvavulopathy in some conditions, early childhood onset in some conditions	Autosomal recessive	*GLA* *LAMP2* *PRKAG2*
Neuromuscular disease [12,29]	Mitochondrial disorders (Friedreich’s ataxia, Barth syndrome)	Disease-specific Friedreich’s ataxia—concentric left ventricular hypertrophy, higher rate of atrial arrhythmia, lower riskof sudden death.	Autosomal recessive, X-linked	*FXN* *TAZ*

HCM: hypertrophic cardiomyopathy; *: compound heterozygosity and homozygosity also identified; ** *FLNC*: variants implicated in various cardiomyopathies, including HCM.
